# The Rise in Homemade Sunscreen Trends and Future Impacts on Skin Cancer Risk: Systematic Review

**DOI:** 10.2196/84694

**Published:** 2026-07-03

**Authors:** Gabi Kaftan, Mia Panillo, Lydia Maxon, Emily Esposito

**Affiliations:** 1 College of Pharmacy and Health Sciences, Sullivan University, 2100 Gardiner Ln, Louisville, KY, 40205, United States, 1 5024138640; 2 College of Osteopathic Medicine, Rocky Vista University, Englewood, CO, United States; 3 St. Olaf College, Northfield, MN, United States

**Keywords:** sunscreen, SPF, homemade, commercial, ultraviolet, ultraviolet radiation, sun protection, social media, UV

## Abstract

**Background:**

The trend of homemade sunscreen recipes has rapidly gained popularity over the last decade, being largely fueled by social media influencers, natural health blogs, and the growing mistrust of large health organizations like the US Food and Drug Administration (FDA). Consumers are increasingly drawn to products labeled “*natural*,” “*organic*,” “*vegan,*” and “*cruelty-free*,” often conflating these terms with safety and effectiveness. However, when applied to sun protection, these assumptions can be dangerously misleading. According to the FDA, there is no verified mathematical formula that can be used to determine an accurate sun protection factor (SPF) rating for the amount of zinc oxide used in many of the recipes found online.

**Objective:**

The objective of this review is to examine the rising popularity and efficacy of homemade sunscreens compared to commercial sunscreens and highlight the potential public health implications of skin cancer risk related to homemade sunscreen use.

**Methods:**

Multiple databases were searched to find current and relevant literature analyzing the social media impact relating to homemade sunscreens and the efficacy of homemade sunscreens compared to commercially available products. Digital content that included or discussed homemade sunscreen recipes was included as well to provide examples of information trending online.

**Results:**

One study analyzing social media trends found that many strong, unverified claims relating to homemade sunscreen versus commercial sunscreen use are being presented in the media, potentially influencing public knowledge. Additional studies analyzing the components of homemade sunscreen recipes compared to components in commercially available products found that homemade sunscreens offer little to no sun protection, therefore increasing the risk of UV damage for individuals using homemade products.

**Conclusions:**

Based on the presented evidence, the adoption of unregulated homemade sunscreens presents a substantial threat to public health. Misinformation spread through social media and influencer culture may unintentionally contribute to an increase in UV-related skin cancers. Greater efforts are needed from health care professionals, regulatory bodies, and online platforms to educate the public and promote the use of scientifically validated sun protection methods.

## Introduction

Skin cancer is the most commonly diagnosed cancer in the United States. Incidence rates for skin cancers continue to rise, especially for basal cell carcinoma (BCC) and squamous cell carcinoma (SCC), which together account for the majority of cases. In the United States, approximately 5.4 million cases of BCC and SCC are diagnosed annually in about 3.3 million individuals, as many people develop multiple tumors [1]. Melanoma is much less common (about 1% of skin cancers), but it is responsible for most skin cancer deaths.

Within the last decade, the popularity of do-it-yourself (DIY) skin care products has overtaken social media due to the rise in fear-mongering over synthetic ingredients deemed “bad” or “harmful.” A post on the popular blog *Wellness Mama* [2] mentions that many sunscreen products contain ingredients such as oxybenzone, which are “endocrine-disrupting chemicals” that “harm ocean life, especially coral.” Statements such as these lead consumers to perceive DIY, “natural,” and/or “organic” sunscreens as safer and more eco-friendly alternatives to commercial products. However, their effectiveness in protecting against harmful UV radiation remains largely unverified. The US Food and Drug Administration (FDA) does not define or regulate “organic” as it applies to skincare, and without FDA regulation, “organic” can be placed on any product without further verification [3].

Sunscreen works to protect from UV radiation by blocking, reflecting, or refracting UV rays from the skin [4]. UVA rays penetrate deeper into the dermis, which induces photoaging, generates reactive oxygen species, and causes DNA damage [5]. UVB primarily penetrates the epidermis and is directly linked to skin cancers and DNA damage [6]. There are two main types of compounds used in sunscreens to block UV radiation, which are physical filters, such as the minerals titanium dioxide and zinc oxide, and chemical filters, such as oxybenzone and avobenzone [4]. Chemical filters are absorbed systemically and absorb high-intensity UV radiation, which excites the chemical compounds to a higher energy state. When the compounds eventually return to their ground state, they then convert the absorbed energy into lower-energy wavelengths, such as infrared radiation. In contrast, physical filters are not absorbed systemically and act as a physical UV barrier that sits on top of the skin.

A study sponsored by the FDA in 2019 found that the blood level of 4 organic compounds surpassed FDA guidelines when sunscreen was applied under maximum use conditions [4]. However, consumers typically do not use this amount of sunscreen, and the study still promoted the use of sunscreen due to its protection from UV rays. The systemic absorption of chemical filters has raised concerns about sunscreen use, especially since animal studies have suggested potential endocrine, reproductive, and neurological toxicities [7]. A study published in 2001 found endocrine-disrupting effects in rats after being administered oral oxybenzone [8]. However, the doses used in this study are essentially unrealistic and unattainable in humans. At this time, there is no strong evidence to suggest negative biological effects from sunscreen.

The homemade sunscreen recipes found throughout the internet all share a common theme: they include different amounts of UV-blocking agents that equate to specific SPF values. For example, *Modern Hippie, Inc* [9] is a health and wellness blog that promotes the use of homemade sunscreen recipes and advises against wearing commercial sunscreens. The blog author claims that 0.27 ounces of zinc oxide equate to an SPF of 2‐5, following the statement that SPF values are not exact, as they are not tested in a laboratory setting. While this conversion seems helpful to users who intend to make their own sunscreen, it is not approved by any formal governing bodies, such as the FDA, and is therefore unreliable in determining safety and efficacy [10].

In contrast to these posts, a 2024 blog post from The Skin Cancer Foundation [11] states, “Homemade formulations that are being shared on social media have no science behind them, and can be dangerous to use.” Commercial sunscreens are considered over-the-counter drugs instead of cosmetic products, which means they must undergo rigorous testing to meet the FDA’s strict criteria. Because of this, the United States only has 16 UV filters approved for use, whereas the European Union has approved 29 filters since sunscreens are labeled as cosmetics [5]. In 2019, the FDA developed generally recognized as safe and effective (GRASE) categories for UV filters, with the 3 categories being category 1: GRASE; category 2: not GRASE; and category 3: insufficient data [7]. Currently, only zinc oxide and titanium dioxide are categorized as GRASE, indicating that these UV filters have undergone a series of clinical investigations that proved them to be noncarcinogenic and without reproductive or developmental risks. These FDA guidelines demonstrate the importance of using FDA-approved sunscreen products for consumer safety.

The clean beauty movement has traded in safe and effective ingredients for an increased risk of skin cancer and contact dermatitis [12]. While it has been proven that the use of a broad-spectrum SPF 30+ decreases the risk for skin cancer, this demographic believes they will develop cancer from FDA-approved sunscreens, not from unprotected sun exposure. This rejection of modern science raises important public health concerns, especially regarding the potential increase in skin cancer risks due to insufficient sun protection. This systematic review aims to analyze the rising popularity, ingredients, and efficacy of DIY sunscreen and highlight the potential risks associated with these non–FDA-approved, homemade sunscreen products.

## Methods

Articles were selected from peer-reviewed journals based on article content, quality, and relevance to the topic of interest. PubMed and Google Scholar were used to find the selected articles using the search terms “homemade sunscreen,” “DIY sunscreen,” and “homemade sunscreen and social media.” Articles were included if they mentioned relevant information regarding the components and/or effectiveness of homemade or commercial sunscreens, or if the article presented information relating to the impacts of homemade sunscreen social media trends. If the article was not available in English, it was excluded. Popular search engines, social media sites, blogs, and other online forums that discussed the production of homemade sunscreens were also searched to provide examples of information relating to homemade sunscreen products being presented in nonscientific or non–peer-reviewed forms. A summary of the inclusion and exclusion criteria is provided in Textbox 1.

The systematic review was conducted based on PRISMA (Preferred Reporting Items for Systematic Reviews and Meta-Analyses) systematic review methodology. All researchers participated in screening and evaluated the suitability of the articles based on the inclusion and exclusion criteria. Titles and abstracts were screened initially by dividing the number of articles in half, with two researchers screening one half of the articles, and two screening the other half, and each pair discussing any discrepancies. For the full-text screening, all authors participated in screening all articles and discussing any discrepancies as a group. Articles were only selected if all authors agreed on inclusion. The completed PRISMA checklist can be found in Checklist 1.

Textbox 1. **Inclusion and exclusion criteria.**

**Inclusion criteria**

- Article content: mentioned relevant information regarding the components and/or effectiveness of homemade or commercial sunscreens. Presented information relating to the impacts of homemade sunscreen social media trends.
- Language: available in English.

**Exclusion criteria**

- Language: not available in English or an English translation.

## Results

### Article Selection

**Figure 1.**
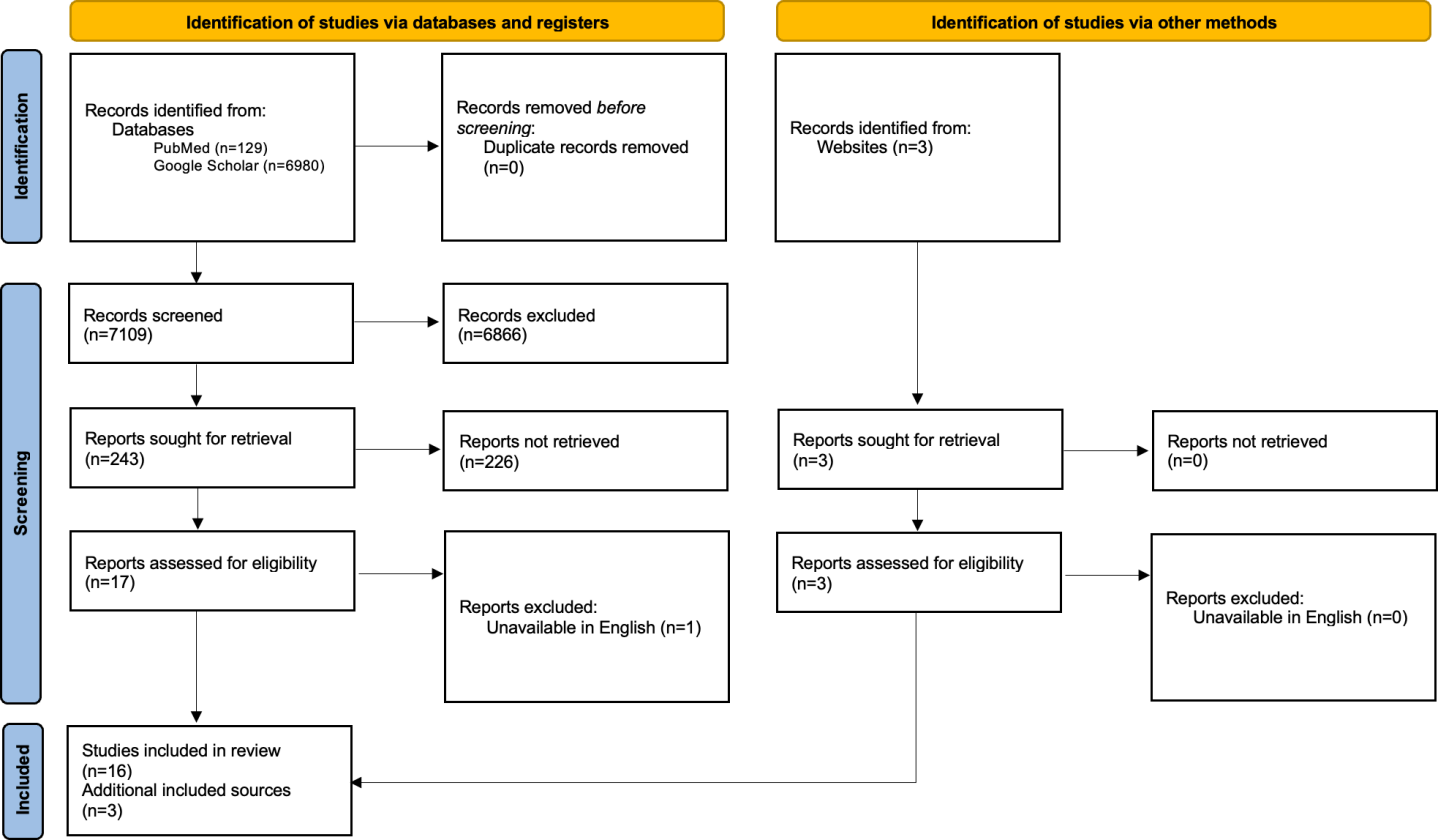
PRISMA (Preferred Reporting Items for Systematic Reviews and Meta-Analyses) flow diagram.

From the search using the specified search terms, 129 articles were identified from PubMed and 6980 were identified from Google Scholar, for a total of 7109 articles (Figure 1). No duplicates were removed in the initial screening. After initial title and abstract screening, the articles were narrowed down to 243 articles, and then to 17 after full article screening based on the inclusion and exclusion criteria. Of the 17 articles selected, one article was excluded because it was not available in English, resulting in 16 peer-reviewed articles being chosen for inclusion. Three online blogs were chosen for additional context relating to our topic.

### Information Being Portrayed by the Media

In a study published in 2020, two researchers coded Pinterest pins via a pilot-tested codebook that had an interrater reliability of 90% [13]. Of the 189 pins included in the study, 95.2% described the effectiveness of homemade sunscreens in a positive manner, and 68.3% of pins recommended recipes for homemade sunscreens that offered insufficient UV protection. In addition, 33.3% of pins made SPF claims ranging from SPF 2 to SPF 50; however, these claims were not backed by evidence-based literature or scientifically verified. This study demonstrates how strong, unverified claims can be presented in the media, potentially influencing public knowledge in a way that may contrast with evidence-based science.

### Comparing the Ingredients Between Homemade Sunscreens and Commercial Sunscreens

Homemade sunscreens are typically made of 4 components: an emollient (oil) base, a photoprotective agent, a water-resistant agent, and essential oils [14]. The emollient base provides a smooth, spreadable component for other ingredients to be mixed into so that the resulting product is easily spreadable on one’s skin. The photoprotective agent is typically an inorganic mineral compound, such as zinc oxide or titanium dioxide, which is the primary UV-blocking component of the recipe that also reflects UV rays as well. The third component usually is a water-resistant agent, such as beeswax, which is included to prevent water loss from the skin and provide environmental protection. Lastly, some recipes include essential oils, which are an optional ingredient that can be added for fragrance or color. A summary of the mentioned homemade sunscreen components, as well as common examples for each, can be found in Table 1.

**Table 1.** Main components of homemade sunscreens.

Homemade sunscreen component	Purpose	Examples
Emollient (oil) base	Creates a smooth base to make the sunscreen easily spreadable onto the skin	Olive oil, coconut oil, almond oil, jojoba oil, carrot seed oil, rose hip seed oil, avocado oil, argan oil, sunflower seed oil
Photoprotective agent	Inorganic minerals added to absorb UV radiation	Zinc oxide, titanium dioxide
Water resistant agent	Prevents dermal water loss and protects against environmental irritants	Beeswax (Cera flava: yellow; Cera alba: white)
Optional: essential oil(s)	Added for fragrance or color	Lavender oil, peppermint oil, tea tree oil, eucalyptus oil, geranium oil

In contrast to homemade sunscreens, FDA-approved sunscreens are typically made of the following components: active ingredients, stabilizing agents, sensory enhancers, and optional ingredients [15]. The active ingredients in the product are the primary UV-filtering agents, and the stabilizing agents are added to the formulation to prevent active ingredient degradation and promote overall shelf stability. More specifically, stabilizing agents such as photostabilizers help prevent degradation of the UV-filtering active ingredients, and emulsifiers help create a stable emulsion between the oil and water phases of the sunscreen, ensuring the ingredients are evenly dispersed throughout the product [15]. Sensory enhancers are compounds that improve the feel or scent of the formula, such as fragrances, moisturizers, and emollients, and they help make application of the product a pleasant experience. The purpose and common examples of the commercial sunscreen components mentioned are summarized in Table 2.

**Table 2.** Main components of commercial sunscreens.

Commercial sunscreen component	Purpose	Examples
Active ingredients	Compounds that filter the sun	Chemical: avobenzone, octinoxate, octisalate, octocrylene, homosalate, oxybenzone Mineral: zinc oxide, titanium dioxide
Stabilizing agents	Photostabilizers, preservatives, thickening agents, and emulsifiers aimed at preventing degradation so that the active ingredients maintain their efficacy and the overall product can remain shelf-stable for long periods of time	Photostabilizers: ethylhexyl methoxycrylene, polyester-7, neopentyl glycol diheptanoate, chelating agents, antioxidants Preservatives: phenoxyethanol, parabens, and formaldehyde-releasing preservatives such as DMDM hydantoin and imidazolidinyl urea Thickening agents: carbomer, cellulose derivatives, acrylates, xanthan gum, agar, arrowroot powder, tapioca starch Emulsifiers: Lecithin, cetearyl alcohol, glyceryl stearate, polysorbates, sorbitan esters, xanthan gum, and beeswax
Sensory enhancers	Ingredients that change the way the product feels or smells	Caprylic/capric triglyceride, squalane, silicones, aloe vera, green tea extract, hyaluronic acid
Optional ingredients	Compounds can be added to provide additional benefits to the sunscreen’s basic sun protection	Aloe, blue-light protectors (ie, iron oxides, zinc oxide, titanium dioxide)

### Efficacy of Homemade Sunscreens

While the recipes for homemade sunscreens seem rather straightforward, consumers fail to understand that when compounded incorrectly, they can degrade the UV component or produce a volatile substance. Certain compounds, such as furocoumarins, bergapten, and psoralens, are phototoxic and contact-sensitizing compounds commonly present in natural oils used for DIY sunscreens [14]. For example, essential oils from *Seseli libanotis* contain furocoumarins that, upon exposure to UV light, generate bergapten—the primary phototoxic compound responsible for photosensitization reactions.

A toxicological study published in 2024 investigated the phototoxicological risks of plant-derived ingredients used in various homemade sunscreen formulations under simulated sunlight [16]. When testing the formulations, many, if not all, of the homemade recipes showed instability and changes in properties such as odor, color, and phase separations when compared to pharmacy-grade or commercially available sunscreens. This ingredient instability can contribute to the development of irritant compounds within the resulting sunscreen, which then can put individuals at a heightened risk for the development of contact dermatitis. The study also found that certain plant-derived components in these DIY recipes did not specify the exact source of herbal ingredients or the need for ingredient standardization in the recipe, and having this standardization of herbal components from correctly identified plant sources is crucial for consistent and reliable effects when using these homemade recipes.

The efficacy of photoprotective compounds, such as zinc oxide, also depends on the particle size and suspension homogeneity of compound particles within the resulting sunscreen [17]. Incorrectly prepared emulsions often create particle aggregates because truly homogeneous suspensions are difficult to create. It is also important to note that these DIY recipes do not use accurate measuring devices that would otherwise be used when compounding in a lab, which can impact the true SPF levels these products are aiming to achieve [18]. Two of the studies selected for this review specifically investigated the efficacy of homemade sunscreens in meeting minimum SPF levels and their ability to protect the skin from UV-induced sun damage.

One study followed the protocols for 15 homemade sunscreen recipes found online, and then analyzed the SPF, the production factor in the UVA domain (PF-UVA), and the critical wavelength of each homemade product in a laboratory setting [18]. The study found that 3 of the products did not create any form of sunscreen with any photoprotective effect whatsoever, and thus carried a major risk of sun damage for users of these recipes. The other 12 recipes resulted in a product that had measured SPF levels of 6 or less, which fails to meet the dermatologist-recommended minimum SPF of 30, therefore carrying a major risk of sun damage for users of these recipes as well.

An additional study conducted in Australia in 2025 compared the efficacy of another natural homemade sunscreen recipe found on a wellness blog to a commercially available SPF 50+ sunscreen [19]. The natural homemade sunscreen contained almond oil, coconut oil, shea butter, beeswax, red raspberry seed oil, carrot seed oil, and zinc oxide. Skin explants were treated with either the homemade product, the commercial SPF 50+ sunscreen, or a base lotion prior to UV exposure, and then assessed via immunohistochemistry for the levels of UV-induced DNA damage and the generation of sunburn cells. The study found that the natural homemade sunscreen was comparable to the commercial sunscreen in reducing UV-induced DNA damage, but it did not effectively protect against the generation of sunburn cells. In comparison, the commercial SPF 50+ sunscreen reduced both UV-induced DNA damage and the generation of sunburn cells. The base lotion provided little to no protection, showing the most UV-induced DNA damage under histological examination and the highest proportion of sunburn cells generated per linear mm of skin.

## Discussion

### Principal Findings

This study found that due to the virality of social media, many claims regarding homemade sunscreen products, which are not backed by scientific evidence, have supported the use of non–FDA-approved products, contributing to misinformation surrounding sun protection. In addition to validating previous studies demonstrating that homemade sunscreen trends promoted online frequently rely on unverified SPF claims and formulations lacking stability, standardization, and regulations, our findings also expand upon prior work by analyzing available social media data and laboratory evidence relating to homemade sunscreen use.

Prior research relating to this topic has established a strong evidence base that, when adequately formulated products are applied and reapplied correctly, both chemical or mineral sunscreen use can significantly reduce an individual’s risk of UV-induced sun damage and sun cancer. Despite this established evidence base, information from unverified blogs and other platforms claiming how commercially available sunscreens contain toxic chemicals that are harmful to both the user and the environment tends to overshadow scientific research [4,7,8]. Available evidence relating to this claim is minimal, as related studies in this review found that unrealistic amounts of commercial sunscreen must be used for any level of toxicity to occur.

Rubin and Brod’s article [12] discusses how claims such as these have contributed to the “clean beauty” movement and led to consumer mistrust of commercially available products. As a result, many consumers have begun to look for more “natural” or “organic” alternatives to commercially available products. As mentioned previously, the FDA does not formally define or regulate the “organic” label for skincare products, which has led to many products claiming to be “organic” without any verification processing. The rise in the use of DIY or homemade sunscreens as a result is largely unregulated, and many homemade recipes involve the use of substances that have not undergone adequate laboratory standardization testing that would ensure the resultant homemade product has adequate product safety and efficacy. Use of homemade recipes can then lead to increased risk of skin cancer and contact dermatitis since the product has not been tested under standardized processes.

When analyzing the alleged SPF against UV radiation in homemade sunscreen, articles included in this review supported that homemade sunscreen products provide minimal to zero efficacy, therefore putting users at a greater risk for developing UV-related skin cancers. The low efficacy of homemade sunscreens may be due to different factors, such as a lack of stabilizing agents to prevent product degradation and improper compounding of ingredients. DIY recipes also lack accurate measuring devices, with influencers equating different amounts of UV-blocking agents to a specific SPF. Without a regulated formulation, the amount of actual UV protection in homemade sunscreens is unknown. Given this inconsistency, individuals who wear homemade sunscreen have unreliable and ineffective protection from the sun. Based on the evidence presented, homemade sunscreens may have an SPF as high as 6, with some providing no SPF at all. Dermatologists recommend sunscreens with an SPF of 30 for adequate sun protection, and if homemade sunscreens do not meet this minimum, then users are at higher risk of experiencing UV damage and developing skin cancers.

Commercially available sunscreens provide significantly higher protection against the generation of sunburn cells in comparison to homemade sunscreens [19]. Commercially available, FDA-approved sunscreens thus offer more reliable and effective sun protection. Additionally, FDA-approved sunscreens undergo rigorous testing and are regulated to ensure safety and UV protection. In comparison, homemade sunscreens lack evidence supporting their efficacy and do not follow regulatory standards, which can lead to inconsistent products. People should use commercially available sunscreens that are scientifically backed by evidence for their overall safety and to ensure they are using a product with verifiable efficacy.

### Limitations

Several limitations are present in this review. For article selection, only articles available in English were selected. This may have excluded key articles that are only available in other languages. There were only a few studies that specifically analyzed the efficacy of homemade sunscreens in a small-scale laboratory setting, therefore limiting the generalizability of our review. Additionally, our research is limited due to narrow search terms. By searching under phrases such as “homemade sunscreen,” “DIY sunscreen,” and “homemade sunscreen and social media,” relevant articles that exclude this terminology may have been missed. The majority of the studies presented in this review examine the chemical properties of homemade sunscreens due to there being a lack of clinical investigations on the efficacy of homemade sunscreens. This further limits the generalizability of the review.

### Future Research

Due to the minimal number of studies that have been published, larger-scale experimental trials would be beneficial in creating a more detailed and generalizable knowledge base of what provides the best sun protection. These studies should include a histological analysis comparing skin reactions after UV exposure on a cellular level between homemade sunscreens and commercial sunscreens, as well as a targeted SPF comparison of UV blocking agents. Additionally, measuring SPF levels among more different homemade sunscreen recipes and seeing how long the homemade products last on the skin compared to commercial products would be helpful as well. Conducting these additional studies could help make the public more well-informed when they are selecting what kind of sunscreen to use and provide more insight into ingredient effectiveness.

### Conclusion

With the rise in DIY sunscreen trends on social media, people need to be wary of whether the information readily available is truly backed by scientific evidence. For the most accurate information, people should seek out peer-reviewed journals and instruction from reputable medical professionals. Social media consumers also need to be willing to verify the information they find online and not blindly trust influencer claims. The increase in misinformation on social media should also raise concerns for physicians and public health officials. Physicians should work to increase education on the dangers of UV damage and preventive measures, such as appropriate sun protection and UV protective clothing, to help combat the influence of social media.

## Supplementary material

10.2196/84694 Checklist 1 PRISMA checklist.

## Abbreviations

BCC: basal cell carcinoma
DIY: do-it-yourself
FDA: Food and Drug Administration
GRASE: generally recognized as safe and effective
PF-UVA: production factor in the UVA domain
PRISMA: Preferred Reporting Items for Systematic Reviews and Meta-Analyses
SCC: squamous cell carcinoma
SPF: sun protection factor
